# *Rice gall dwarf virus* exploits tubules to facilitate viral spread among cultured insect vector cells derived from leafhopper *Recilia dorsalis*

**DOI:** 10.3389/fmicb.2013.00206

**Published:** 2013-07-23

**Authors:** Hongyan Chen, Limin Zheng, Dongsheng Jia, Peng Zhang, Qian Chen, Qifei Liu, Taiyun Wei

**Affiliations:** Fujian Province Key Laboratory of Plant Virology, Institute of Plant Virology, Fujian Agriculture and Forestry UniversityFuzhou, China

**Keywords:** rice gall dwarf virus, leafhopper vector *Recilia dorsalis*, tubules, viral spread, continuous cell cultures of leafhopper

## Abstract

*Rice gall dwarf virus* (RGDV), a member of the family *Reoviridae*, causes repeated epidemics in rice fields in southern China. An RGDV isolate collected from Guangdong Province (southern China) is mainly transmitted by leafhopper vector *Recilia dorsalis* in a persistent-propagative manner. The infection by RGDV induces the formation of virus-containing tubules in the plant host and insect vector. In this study, we established continuous cell cultures of the leafhopper *R. dorsalis* to investigate the functional role of these tubules within the insect vector. Cytopathologic studies revealed that the tubules, which comprised viral non-structural protein Pns11 and contained viral particles, were able to protrude from the surface of cultured leafhopper cells. Tubule-like structures formed in non-host insect cells after the expression of Pns11 in a baculovirus system, suggesting that Pns11 was the minimal viral factor required for the formation of the tubules induced by RGDV infection. In cultured leafhopper cells, knockdown of Pns11 expression from RNA interference, induced by synthesized dsRNA from the Pns11 gene, abolished the formation of such tubules, preventing the direct cell-to-cell spread of RGDV without significant effects on viral multiplication. All these results show that RGDV exploits virus-containing tubules to facilitate viral spread among its insect vector cells.

## Introduction

*Rice gall dwarf virus* (RGDV), *Rice dwarf virus* (RDV) and *Wound tumor virus*, members of the genus *Phytoreovirus* in the family *Reoviridae*, are transmitted propagatively to plants by leafhopper vectors (Boccardo and Milne, 1984). Rice gall dwarf disease induced by RGDV was first recorded in Thailand in 1979 and was later discovered in China and Southeast Asia (Omura et al., 1980; Putta et al., 1980; Ong and Omura, 1982; Fan et al., 1983). Since 2000, the disease has repeatedly affected rice fields in southern China (Zhang et al., 2008). RGDV from Guangdong Province in southern China is mainly transmitted by a leafhopper vector, *Recilia dorsalis*, in a persistent-propagative manner (Fan et al., 1983). By 2012, the disease had spread to other areas of Guangdong Province, and the leafhopper vector *R. dorsalis* developed a higher affinity with RGDV. RGDV thus has the potential to become one of the greatest threats to rice production in these regions.

RGDV is an icosahedral double-layer particle approximately 65–70 nm in diameter, with a 12-segmented dsRNA genome (Moriyasu et al., 2000, 2007; Miyazaki et al., 2005; Zhang et al., 2008). Six segments (S1, S2, S3, S5, S6, and S8) encode structural proteins (P1, P2, P3, P5, P6, and P8), and the remainder encode non-structural proteins (Pns4, Pns7, Pns9, Pns10, Pns11, and Pns12) (Moriyasu et al., 2000, 2007; Zhang et al., 2008). Among the structural proteins, P3 is the core capsid protein, which encloses P1, P5, and P6 (Omura et al., 1985; Ichimi et al., 2002), and P2 and P8 are the minor and major outer capsid proteins, respectively (Omura et al., 1998; Miyazaki et al., 2005). Among the non-structural proteins, Pns7, Pns9, and Pns12 are the components of the viroplasm, the site for viral replication and assembly (Wei et al., 2009, 2011; Akita et al., 2011), Pns11 is a viral RNA-silencing suppressor (Shen et al., 2012), and the functions of Pns4 and Pns10 are unknown.

RGDV must replicate and spread within the insect body of the leafhopper *R. dorsalis* to be transmitted to the plant host. Previous cytopathological studies of phytoreovirus in infected plants and vector insects characterized two kinds of inclusions: the viroplasm and tubules (Fukushi et al., 1962; Omura et al., 1985). With the use of cultured monolayers of insect vector cells, the induction of the viroplasm by RGDV infection has been examined in detail (Wei et al., 2009, 2011; Akita et al., 2011); however, the mechanism underlying the genesis and maturation of the tubules is unknown. Pns11 of RGDV corresponds to Pns10 of RDV, which is the component of the tubules (Moriyasu et al., 2000; Wei et al., 2006). Pns10 of RDV has been demonstrated to facilitate viral spread among cultured insect vector cells (Wei et al., 2006, 2008) and the spread of RDV in the body of its leafhopper vector (Chen et al., 2012). Whether Pns11 of RGDV is similarly involved in tubule formation and viral spread within its insect vector is still unknown.

In the present study, we developed continuous cell cultures of leafhopper *R. dorsalis* to investigate the functional role of the tubules induced by RGDV in its insect vector. Cytopathologic results showed that viral non-structural protein Pns11 was the minimal viral factor required for the formation of the tubules induced by RGDV infection. Such tubules protruded from the infected cell surface and attached to adjacent uninfected cells. The knockdown of Pns11 expression due to RNA interference (RNAi) induced by synthesized dsRNA from Pns11 gene abolished the formation of the tubules, preventing direct cell-to-cell spread of RGDV without significant effects on the multiplication of RGDV. All these results indicated that RGDV exploited virus-containing tubules to facilitate viral spread among insect vector cells. These results will promote our understanding of the mechanism underlying the spread of RGDV within its insect vector.

## Materials and methods

### Virus and antibodies

RGDV samples, collected from rice fields from Guangdong Province, China, were maintained on rice plants via transmission by *R. dorsalis*. RGDV was purified from infected rice plants and stored at −80°C as described previously (Omura et al., 1982). Polyclonal antiserum against P8, Pns7 and Pns11 of RGDV were prepared in rabbits as described previously (Moriyasu et al., 2000, 2007; Wei et al., 2009). IgGs were purified from the antibody sample using a protein A-Sepharose affinity column and then conjugated directly to fluorescein isothiocyanate (FITC) or rhodamine according to the manufacturer's instructions (Invitrogen).

### Establishment of continuous cell cultures derived from leafhopper *R. dorsalis* for viral infection

The cell line of the leafhopper *R. dorsalis* was established by adapting the protocol described by Kimura and Omura (1988). Primary cell cultures of *R. dorsalis*, originally established from the embryonic fragments dissected from eggs of *R. dorsalis*, were maintained in monolayer culture at 25°C in Kimura's insect medium as described previously (Kimura and Omura, 1988). Such vector cells in monolayers (VCMs) were then transferred into culture flasks for further subculturing.

The purifed RGDV was used to inoculate VCMs on coverslips that had been washed with 0.1 M histidine that contained 0.01 M MgCl_2_, pH 6.2 (His-Mg), as described previously (Wei et al., 2011).

### Baculovirus expression of non-structural proteins of RGDV

The baculovirus system was used to express non-structural proteins Pns11 and Pns7 of RGDV, using the protocol described by Wei et al. (2006). Recombinant baculovirus vectors containing Pns11 or Pns7 were introduced into DH10Bac (Invitrogen) for transposition into the bacmid. Sf9 cells were infected with recombinant bacmids in the presence of Cellfectin (Invitrogen).

### Immunofluorescence staining

VCMs or Sf9 cells on coverslip were fixed in 4% paraformaldehyde, immunolabeled with Pns11-specific IgG that had been conjugated to FITC (Pns11-FITC), Pns7-specific IgG that had been conjugated to FITC (Pns7-FITC), or major outer capsid protein P8-specific IgG that had been conjugated to rhodamine (P8-rhodamine) and then processed for immunofluorescence, as described previously (Wei et al., 2006, 2011). The cells were observed with a Leica TCS SP5 laser confocal microscope.

### Electron microscopy

VCMs or Sf9 cells on coverslips were prepared for transmission electron microscopy as described previously (Wei et al., 2006, 2009, 2011). For immunoelectron microscopy, the cell sections were immunolabeled with the Pns11-specific IgG as the primary antibody, followed by treatment with and goat anti-rabbit IgG conjugated with 15- or 10-nm gold particles, as secondary antibodies (Sigma), as described previously (Wei et al., 2006).

### Examination of RGDV spread and infection of VCMs in the presence of synthesized dsRNAs

We designed primers for PCR amplification of a 1071-bp segment of the Pns11 gene of RGDV and a 717-bp segment of a green fluorescence protein (GFP)-encoding gene as a control. The PCR products were used for dsRNA synthesis according to the protocol of the T7 RiboMAX Express RNAi System kit (Promega). For examining the effects of synthesized dsRNAs on viral spread of RGDV, VCMs were transfected with 0.5μg/μL dsRNAs in the presence of Cellfectin (Invitrogen) for 8 h. VCMs were then inoculated with RGDV at a low multiplicity of infection (MOI) of 0.001 or a high MOI of 10 in the presence of virus-neutralizing antibodies (30 mg/mL of medium), as described previously (Wei et al., 2006). VCMs were fixed 3 h post inoculation (hpi), immunolabeled with Pns11-FITC and P8-rhodamine and visualized by fluorescence microscopy. The fluorescent cells were counted by the focus count method (Kimura, 1986). In this method, an infected cell and any adjoining infected cells were counted as one infectious unit. A minimum of four fields was counted for each sample from three or more independent experiments.

To confirm whether the synthesized dsPns11 affected multiplication of RGDV, VCMs were transfected with 0.5μg/μL dsRNAs in the presence of Cellfectin for 8 h and then inoculated with RGDV at an MOI of 10. At 72 hpi, proteins were extracted from infected cells and further analysed by SDS-PAGE and immunoblotting with Pns11-specific or P8-specific antibodies, respectively. Insect actin was detected with actin-specific antibodies as a control to confirm loading of equal amounts of proteins in each lane.

## Results

### Tubules were formed by non-structural protein pns11 of RGDV in insect vector cells

**Figure 1:**
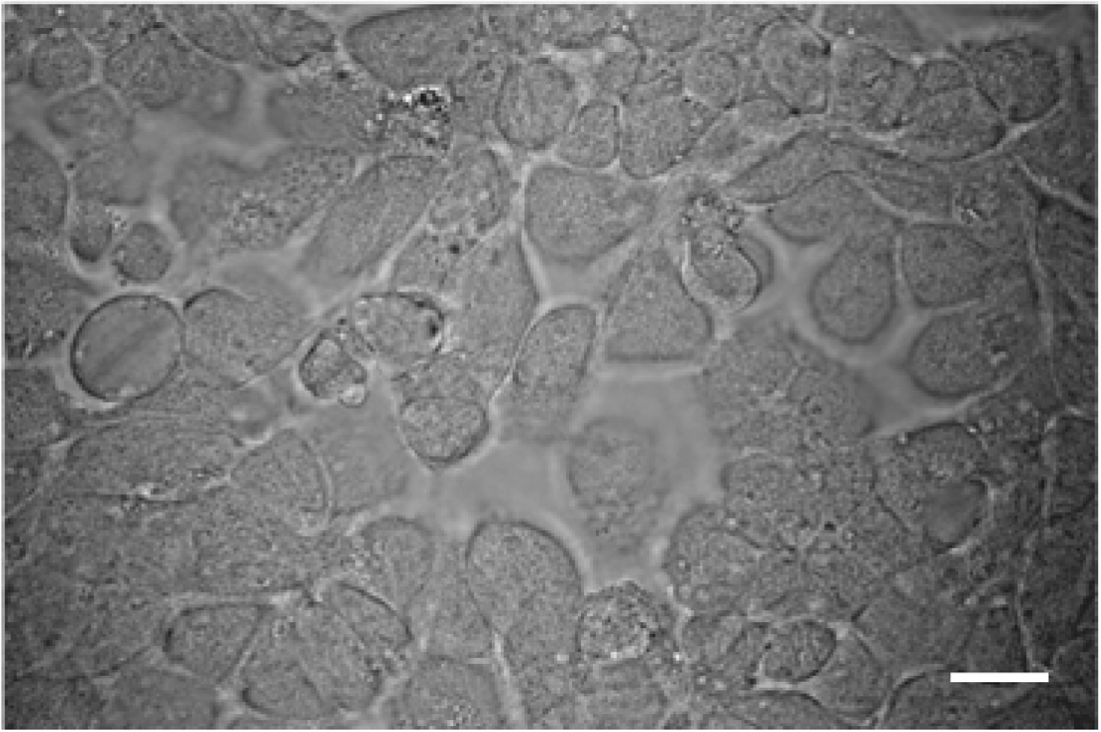
**Light micrograph of *R. dorsalis* vector cells in monolayer (VCM) after 90 passages. Bars, 50μm**.

**Figure 2:**
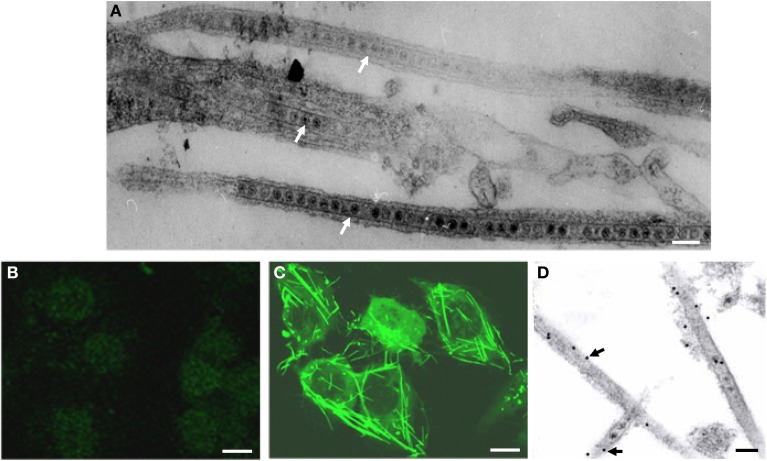
**Viral non-structural protein Pns11 is the constituent of the tubules induced by RGDV infection in VCMs at 48 hpi. (A)** Electron micrograph of virus-containing tubules within the cytoplasm or protruding from the surface of infected cells. Bar, 100 nm. **(B)** Immunofluorescence staining of Pns11 showing nothing within uninfected cell. VCMs were immunostained with Pns11-FITC. Bar, 10μm. **(C)** Immunofluorescence staining of Pns11 showing tubule-like structures within the cytoplasm and protruding from the surface of infected cell. VCMs were immunostained with Pns11-FITC. Bar, 10μm. **(D)** Immunogold labeling of Pns11 in the tubules in RGDV-infected VCMs. Cells were immunostained with Pns11-specific IgG as primary antibodies, followed by treatment with 15-nm gold particle-conjugated goat antibodies against rabbit IgG as secondary antibodies. White arrows mark virus-containing tubules. Black arrows mark gold particles. Bar, 100 nm.

A leafhopper cell line, originating from embryonic fragments dissected from eggs of *R. dorsalis*, was established. After 90 passages of subculturing at 7-day intervals, the dominant cell type in the established *R. dorsalis* cell line was epithelial-like and approximately 35–60μm in diameter (Figure 1). Examination of the thin sections of RGDV-infected VCMs at 48 hpi by electron microscopy revealed virus-containing tubules approximately 85 nm in diameter within the cytoplasm or protruding from the surface of leafhopper cells (Figure 2A). These virus-containing tubules sometimes extended into the cell protrusions, namely, filopodia, from the infected cells, suggesting that RGDV particles were accompanied by the tubules to pass through the filopodia of infected cells. The non-structural protein Pns11 of RGDV was found in numerous tubule-like structures within the cytoplasm or protruding from the cell surface (Figure 2C). When the subcellular localization of Pns11 of RGDV in infected VCM was examined by immunoelectron microscopy, the tubules were specifically immunolabeled with Pns11 antibodies (Figure 2D), confirming that Pns11 was a component of the tubules.

**Figure 3:**
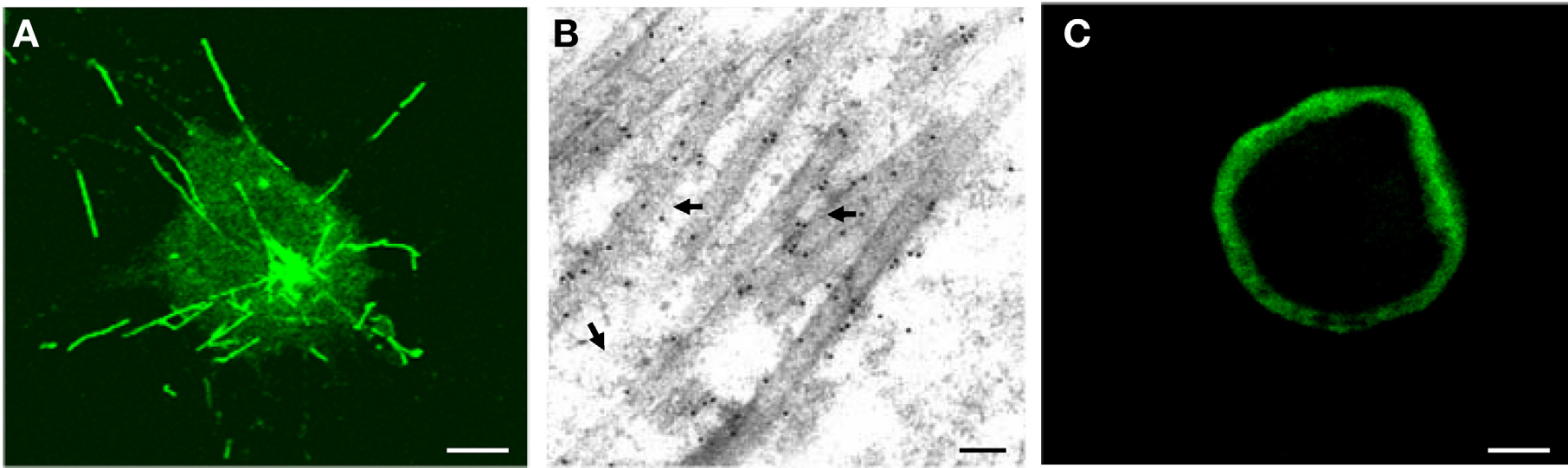
**Subcellular localization of Pns11 in recombinant baculovirus-infected Sf9 cells at 72 hpi. (A)** Immunofluorescence staining of Pns11 showing tubule-like structures within the cytoplasm or protruding from the cell surface. Sf9 cells were immunostained with Pns11-FITC. Bar, 10μm. **(B)** Immunogold labeling of Pns11 in the tubules. Cells were immunostained with Pns11-specific IgG as primary antibodies, followed by treatment with 10-nm gold particle-conjugated goat antibodies against rabbit IgG as secondary antibodies. Arrows mark gold particles. Bar, 100 nm. **(C)** Immunofluorescence staining of Pns7 showing the diffuse distribution of Pns7 in the cytoplasm of cell. Sf9 cells were immunostained with Pns7-FITC. Bar, 10μm.

To determine whether Pns11 of RGDV had an inherent ability to form the tubules, we used a baculovirus system to express Pns11 in Sf9 cells. As seen by immunofluorescence microscopy, Pns11 was distributed in the tubule-like structures in the cytoplasm or protruding from the cell surface (Figure 3A). Immunoelectron microscopy confirmed that Pns11-specific IgG reacted specifically with the tubules approximately 85 nm in diameter (Figure 3B). However, Pns7 of RGDV was distributed diffusely throughout the cytoplasm (Figure 3C). All these results demonstrated that virus-containing tubules in infected insect vector cells were basically formed by non-structural protein Pns11 of RGDV.

### The tubules protruded from initially infected cells and attached to adjacent uninfected cells

To study the distribution of the tubules over time, we used immunofluorescence microscopy to visualize the tubules in the VCMs during infection by RGDV. VCMs were inoculated with RGDV at a low MOI of 0.001 for 2 h and then cultured in the presence of virus-neutralizing antibodies to prevent infection by free viral particles. At this low MOI, the initial and secondary infection of RGDV in VCMs could be easily monitored (data not shown).

**Figure 4:**
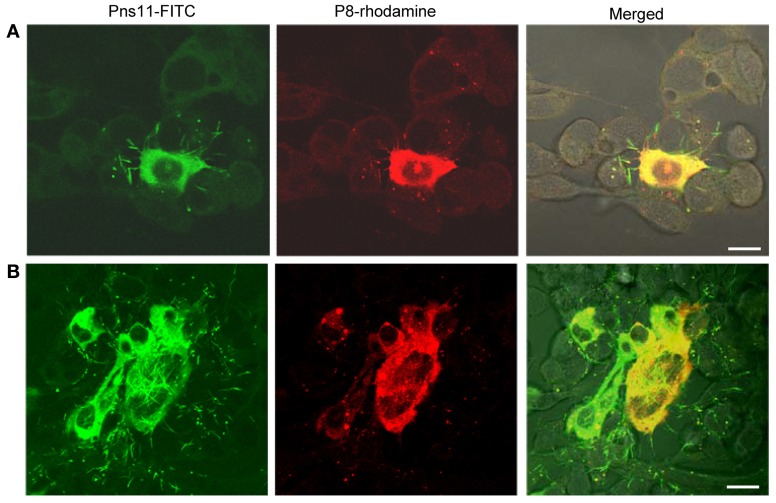
**Subcellular localization of the tubules and P8 antigens of RGDV in virus-infected VCMs viewed at 24 (A) and 72 (B) hpi.** VCMs were inoculated with RGDV at an MOI of 0.001 and cultured in the presence of virus-neutralizing antibodies. VCMs were immunostained with Pns11-FITC and P8-rhodamine and viewed with confocal fluorescence microscopy. Images are representative of multiple experiments with multiple preparations. Bars, 10μm.

As early as 24 hpi, small foci of one or two infected cells were visible in the presence of virus-neutralizing antibodies (Figure 4A). At 72 hpi, RGDV appeared to have spread from the initially infected cells to adjacent uninfected cells to form infection foci of 9–13 cells in the presence of virus-neutralizing antibodies (Figure 4B). The tubules were protruding from or scattered outside the infected cells, even near the surface of adjacent uninfected cells (Figure 4). All these results suggested that RGDV might spread from initially infected cells to adjacent uninfected cells by exploiting the tubules.

### RNAi induced by dsRNA from pns11 gene significantly inhibits formation of tubules and viral spread among insect vector cells

**Figure 5:**
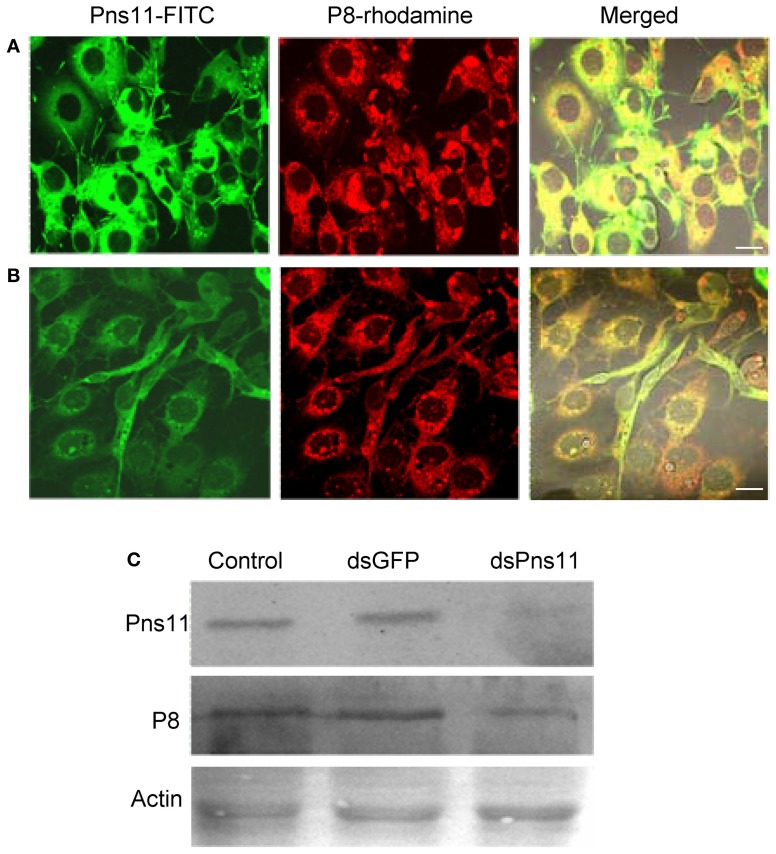
**RNAi induced by dsPns11 inhibited the formation of the tubules without significant effects on RGDV multiplication in VCMs.** Twenty-four hours after transfection with dsGFP **(A)** or dsPns11 **(B)**, VCMs were inoculated with RGDV at an MOI of 10 and cultured in the presence of virus-neutralizing antibodies. At 72 hpi, cells were immunolabeled with Pns11-FITC and P8-rhodamine, then examined with confocal microscopy. Images are representative of multiple experiments with multiple preparations. Bars, 30μm. **(C)** RNAi induced by dsPns11 significantly reduced the expression of Pns11 but not P8 of RGDV in virus-infected VCMs as shown here in Western blots. Protein extracts from cells transfected with Cellfectin, dsGFP or dsPns11 were separated by SDS-PAGE to detect Pns11 or P8 with Pns11-specific or P8-specific antibodies, respectively. Insect actin was detected with actin-specific antibodies as a control.

For examining whether RNAi induced by dsRNAs affected viral multiplication in VCMs, VCMs were transfected with the synthesized dsRNAs targeting the encoding sequences of Pns11 (dsPns11) or GFP (dsGFP). At 8 h after transfection, VCMs were inoculated with RGDV at an MOI of 10, and then fixed at 72 hpi and immunolabeled with Pns11-FITC and P8-rhodamine. In VCMs transfected with dsGFP, viral infection was observed in almost 100% of cells, and abundant tubules protruded from the infected cell surface (Figure 5A). In VCMs transfected with dsPns11, the number of infected VCMs was not significantly reduced, but the formation of tubules was significantly inhibited (Figure 5B), suggesting that the expression of Pns11 had been knocked down by RNAi induced by dsPns11. To determine the effects of RNAi induced by dsPns11 on the expression of Pns11, we collected cell lysates for assay by Western blot. The expression of Pns11 and P8 under dsGFP transfection was the same as the control treatment (Figure 5C). However, transfection with dsPns11 resulted in a significant reduction in the level of Pns11 expression, but only weakly reduced the expression level of P8 (Figure 5C). Our results suggested that the inhibition of tubule formation by treatment with dsPns11 might not significantly affect viral multiplication in VCMs.

**Figure 6:**
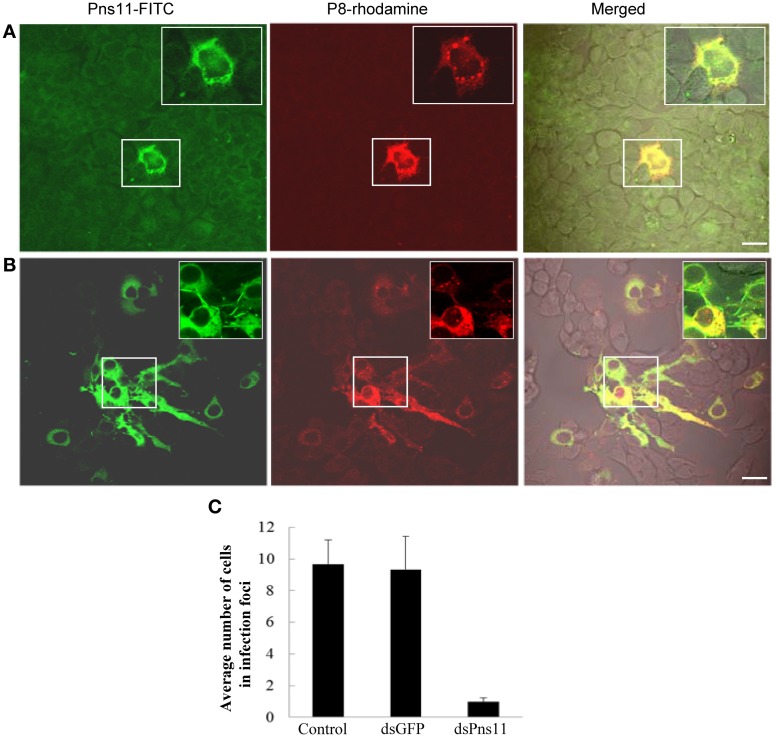
**RNAi induced by dsPns11 inhibited the spread of RGDV among insect vector cells.** At 24 h after transfection with dsPns11 **(A)** or dsGFP **(B)**, VCMs were inoculated with RGDV at a low MOI (0.001) and cultured in the presence of virus-neutralizing antibodies. At 72 hpi, cells were immunolabeled with Pns11-FITC and P8-rhodamine, and then examined by confocal microscopy. Bars, 15μm. Insets are enlarged images of boxed areas. **(C)** Mean number of RGDV-infected cells/infection focus in VCMs after treatment with dsRNAs. Error bars indicate standard deviations from three independent experiments.

To determine whether the treatment of dsPns11 inhibited viral spread among insect vector cells, 8 h after transfection with dsRNAs, we inoculated VCMs with RGDV at a low MOI of 0.001, cultured in the presence of virus-neutralizing antibodies and then processed for immunofluorescence. At 72 hpi, viral infection was seen in the infection foci with approximately 9-11 infected cells in VCMs that received dsGFP and Cellfectin reagent (Figures 6B,C, data not shown). However, in VCMs that received dsPns11, viral infection was restricted to one or two cells, and the formation of tubules was inhibited (Figures 6A,C). Because VCMs treated with dsPns11 were able to support normal viral multiplication, we deduced that the limited infection of RGDV in VCMs is caused specifically by the inhibition of the intercellular spread of RGDV resulting from the failure of the tubules to form after treatment with dsPns11. Taken together, our results suggested that RGDV exploits the tubules to spread among insect vector cells.

## Discussion

During RGDV infection of the leafhopper *R. dorsalis* vector cells grown in monolayers (VCMs) in the present study, virus-containing tubules of approximately 85 nm in diameter were formed (Figure 2) and comprised viral non-structural protein Pns11 (Figure 2). When Pns11 of RGDV was expressed alone, such tubules formed in non-host Sf9 cells (Figure 3), suggesting that Pns11 was the minimal viral factor required for the formation of the tubules induced by RGDV infection.

As we noted earlier, Pns11 of RGDV corresponds to Pns10 of RDV, the component of the tubule induced by RDV infection (Moriyasu et al., 2000). Because the tubules constructed by Pns10 of RDV are directly involved in the intercellular spread of RDV particles among cultured leafhopper cells (Wei et al., 2006, 2008; Pu et al., 2011), here, we examined whether the tubules induced by RGDV infection also facilitate viral spread among insect vector cells. By 72 hpi, RGDV particles had spread from initially infected cells to adjacent uninfected cells, forming larger infection foci in the presence of neutralizing antibodies to avoid the infection by free viral particles (Figure 4), and the tubules protruded from the infected cell surface (Figure 4). Because viral particles were accompanied by the tubules, we deduced that RGDV might exploit the tubules to spread among insect vector cells. To confirm this hypothesis, we used an RNAi strategy to specifically knock down the expression of Pns11 by treatment with dsPns11; the tubules failed to assemble, and viral spread was prevented in the VCMs without significant effects on viral multiplication (Figures 5, 6). Taken together, all these results indicated that the tubules facilitated viral spread among insect vector cells. Tubules were associated with the actin-based cell protrusion, namely, filopodia, in the infected cells (Figure 2A). We deduced that the tubules might exploit the actin-based filopodia to attach to healthy adjacent cells for viral direct intercellular spread. Recently, we also showed that such tubules could facilitate the spread of RDV in the body of its leafhopper vector (Chen et al., 2012). Whether the tubules induced by RGDV infection have similar functions in their insect vectors will be investigated in the future.

We also previously demonstrated that the transport of RGDV particles from viroplasms, the site for viral replication and assembly, to the plasma membrane and into the medium is dependent on the microtubules of the infected VCMs (Wei et al., 2009). Infection by these free viral particles in the medium was protected by the addition of neutralizing antibodies. Furthermore, we have shown that RGDV particles were accompanied by the tubules and spread from infected cells to uninfected cells in the presence of neutralizing antibodies. The model for viral direct intercellular spread presented here is thought to be advantageous over infection by cell-free virus because the virus is protected from host immune responses. Thus, RGDV evolved to exploit two pathways for the release viral particles from infected vector cells, without affecting cellular structures or morphology, corresponding to the evidence that the leafhopper cell line supports a non-cytopathic, persistent infection of RGDV. Evidently, mechanisms have evolved in the vector cells to allow efficient release of RGDV without any significant pathology.

### Conflict of interest statement

The authors declare that the research was conducted in the absence of any commercial or financial relationships that could be construed as a potential conflict of interest.
